# Prayer of Socrates

**Published:** 1889-04

**Authors:** 


### PRAYER OF SOCRATES.

"O beloved Pan, and all ye other gods of this place, grant me to become beautiful in the inner man, and that whatever outward things I

have may be at peace with those within. May I deem the wise man rich, and may I have such a portion of gold as none but a prudent man can either bear or employ."

